# Typologies of Conflict, Migration, and Sexual Health Risk among Female Sex Workers in Ukraine Post-Russian Invasion: A Latent Class Analysis

**DOI:** 10.21203/rs.3.rs-11078270/v1

**Published:** 2026-10-13

**Authors:** Marcela Lopez, Cho-Hee Shrader, Pavlo Smyrnov, Daniel M. Parker, Tetyana I. Vasylyeva

## Abstract

Background In February 2022, the Russian government launched a full-scale invasion of Ukraine, displacing millions. To better understand how migration, conflict, and exposure to violence may have shaped risk perception and behaviors among female sex workers (FSWs) in Ukraine, we sought to typologize trends in migration trajectories and past experiences among relocated FSWs and to compare their sexual health behaviors and perceptions with those of non-relocated FSWs. Methods A peer-driven intervention conducted by the Alliance for Public Health interviewed individuals engaging in paid sex work in Ukraine, from September 2023 to January 2024. We conducted a latent class analysis to typologize groups of the interviewed women who had relocated since the Russian invasion based on shared migration experiences, histories of displacement, and past exposures to violence. We used logistic regression to compare post-invasion initiation into sex work, perceptions of sexual risk, and inconsistent condom use between our selected classes and non-relocated FSWs. We used negative binomial regression to compare their reported numbers of sexual partners. Results A total of 2,055 FSWs were interviewed. Two latent classes fit our data best: one defined as those who had experienced conflict related adversity (Class 1) and another based on personal adversity (Class 2). Compared to non-relocated FSWs, women in Class 1 had greater odds of not having engaged in sex work prior to the invasion (aOR: 14.00; 95% CI: 7.83–27.43), greater odds of reporting their sexual behavior becoming riskier since the invasion (aOR: 1.57; 95% CI: 1.13–2.18), decreased odds of inconsistent condom use (aOR: 0.54; 95% CI: 0.38–0.76), and more sexual partners (aRR: 1.14; 95% CI: 1.05–2.23); while women from Class 2 had increased odds of inconsistent condom use (aOR: 2.83; 95% CI: 1.72–4.66), and fewer sexual partners (aRR: 0.90; 95% CI: 0.82–0.99). Conclusion Factors that define the type of adversity experienced by relocated women, like the intensity of conflict in their former cities of residence or distance traveled, may be precursors to riskier sexual behavior. Self-reported displacement may not fully capture an individual’s sexual risk profile. Interventions designed to minimize risks for women engaging in sex work should consider their individual migration histories.

